# Efficient Conversion of Lignin Waste to High Value Bio-Graphene Oxide Nanomaterials

**DOI:** 10.3390/polym11040623

**Published:** 2019-04-04

**Authors:** Jinghao Li, Qiangu Yan, Xuefeng Zhang, Jilei Zhang, Zhiyong Cai

**Affiliations:** 1 U.S. Department of Agriculture, Forest Service, Forest Products Laboratory, Madison, WI 53726, USA; csuftljh@gmail.com; 2 Ligsteel LLC, Madison, WI 53726, USA; 3 Department of Sustainable Bioproducts, Mississippi State University, Mississippi State, MS 39762, USA; xz210@msstate.edu (X.Z.); jz27@msstate.edu (J.Z.)

**Keywords:** Kraft lignin, lignin graphite, lignin graphene oxide, reduced lignin graphene oxide, characterization, applications

## Abstract

Lignin graphene oxide was oxidized after Kraft lignin was graphitized by thermal catalytic conversion. The reduced lignin graphene oxide was derived from lignin graphene oxide through thermal reduction treatment. These Kraft lignin, lignin graphite, lignin graphene oxide, and reduced lignin graphene oxide were characterized by scanning electron microscopy, raman microscopy, high-resolution transmission electron microscopy, X-ray diffraction, Fourier transform infrared spectroscopy, atomic force microscopy and thermogravimetric analysis. The results showed lignin graphite converted from Kraft lignin had fewer layers with smaller lateral size than natural graphite. Moreover, lignin graphene oxide was successfully produced from lignin graphite by an oxidation reaction with an hour-long reaction time, which has remarkably shorter reaction time than that of graphene oxide made from natural graphite. Meanwhile, this lignin-derived graphene oxide had the same XRD, FTIR and Raman peaks as graphene oxide oxidized from natural graphite. The SEM, TEM, and AFM images showed that this lignin graphene oxide with 1–3 average layers has a smaller lateral size than that of graphene oxide made from natural graphite. Moreover, the lignin graphene oxide can be reduced to reduced lignin graphene oxide to fabricate graphene-based aerogel, wire, and film for some potential applications.

## 1. Introduction

Lignin, as a component in the cell walls of plants, is used to strengthen their structure, and it is the most abundant aromatic biopolymer on Earth [1]. There are several different types of lignin raw materials produced by different methods, which exhibit distinct properties for niche applications. Lignosulfonates, one of several common lignin raw materials, have a wide variety of applications such as dispersed pesticides, dyes, carbon black, plasticizers and chemicals [2]. Currently, lignosulfonates account for 90% of the total commercial lignin market with annual worldwide production of approximately 1.8 million tons [3]. Compared to lignosulfonates, Kraft lignin is the largest raw lignin byproduct produced from the Kraft pulping process, which produces approximately 50–70 tons per year, worldwide [3]. However, the utilization of Kraft lignin is limited, and an estimated 98% of Kraft lignin is burned on site, after the pulping process, as a non-optimized energy resource to provide steam power [4,5]. Therefore, researchers have been trying to valorize Kraft lignin for value-added chemicals or materials suitable for large-scale industrial applications [6].

Graphene is an exciting material that has a one-atom-thick 1D planar sheet of sp^2^-bonded carbon atoms in a honeycomb crystal lattice structure displaying excellent electrical and mechanical properties [7]. In recent decades, graphene materials attracted significant attention due to their potential applications in transistors, conductors, batteries, catalysts, and biosensors [8,9,10,11,12]. Nowadays, graphene can be produced by mechanical exfoliation, epitaxial growth and chemical vapor deposition [13]. However, it has been difficult to scale up production for high volume applications due to low yields and high costs. Recently, researchers developed a new method of converting Kraft lignin to graphene-based materials by catalytic graphitization [14]. The effects of temperature, catalysts, particle size, material properties and material characteristics have been extensively analyzed. These experiments demonstrated that high-quality nano-size multi-layer bio-graphene materials, with similar properties to current graphene products, can be successfully produced by a low cost thermal conversion process. Moreover, this method can be efficiently scaled up to produce large amounts of graphene material with high yields from byproduct lignin raw materials. However, this derived-graphene is strongly hydrophobic and highly stable which may not facilitate the assembly of macroscopic structures by simple solution processes [15]. Graphene oxide (GO), generally produced using natural graphite with cost-effective chemical methods, has some important characteristics [15]. This material, with hydrophilic groups, can be modified and reacted with other materials or additives to assemble graphene-based composites for many applications by a simple solution process. As a result, both GO and reduced graphene oxide are hot research topics in recent years.

In this study, the Kraft lignin, as the carbon source, was used to make few-layered lignin graphite (LG), lignin graphene oxide (LGO) and reduced lignin graphene oxide (RLGO). The morphology, structure, characteristics and properties of LGO and its relative materials (Kraft lignin, LG, RLGO) were examined by a scanning electron microscope (SEM), transmission electron microscopy (TEM), atomic force microscopy (AFM), Fourier-transform infrared spectroscopy (FTIR), X-ray diffraction (XRD), Raman spectroscopy and thermogravimetric analysis (TGA). The applications of LGO/cellulose nanofibrils (CNF) aerogel, LGO/CNF film, LGO/polyacrylonitrile (PAN) electrical wire, and LGO/PAN fiber sheet fabricated using this synthesized LGO were also developed in this study.

## 2. Experimental Section

### 2.1. Chemicals and Materials

Iron(III) nitrate nonahydrate (Fe(NO_3_)_3_·9H_2_O), tetrahydrofuran (THF), potassium permanganate (KMnO_4_), and sodium nitrate (NaNO_3_) were purchased from Sigma-Aldrich (St. Louis, MO, USA); hydrogen peroxide (H_2_O_2_) was obtained from Fisher scientific (Hampton, NH, USA). Commercial Kraft lignin named Bio-Choice lignin was provided by Domtar Corporation (Fort Mill, SC, USA), the specification provided by Domtar showed that the Kraft lignin contained 97.1% lignin and 1.7% sugar with a pH value of 6.2. The content of C, H, and O was 65.9%, 7.5%, and 26.3%, while proximate analysis showed the content of volatile, fixed carbon, and ash as 54.5%, 43,1%, and 2.4%, respectively [14,16]. The element analysis results and more characterizations of raw Kraft lignin was presented in our previous research [17].

### 2.2. Preparation and Reaction Process

Kraft lignin used in this study as a carbon source was BioChoice Lignin supplied by Domtar Corporation. Iron (III) nitrate was used in this study as metal catalyst source for the catalytic graphitization. Iron with 10% loading in the iron-lignin precursors was prepared at room temperature using a co-precipitation technique. Specifically, lignin solution was prepared by adding 50 g of Kraft lignin to 50 mL tetrahydrofuran (THF) in a 250-mL glass beaker and stirring the mixture for 2 h. Meanwhile, iron nitrate solution was prepared by adding 41 g of iron (III) nitrate nonahydrate to 20 mL DI water in a 100-mL glass beaker and stirring the mixture until the metal salt dissolved completely. Prepared iron nitrate solution drop-like (~10 mL/min) was added to the lignin solution, and the lignin-iron nitrate mixture was stirred for 2 h at 70 °C. The final iron-lignin mixture was kept at room temperature for 24 h, and then transferred to an oven and dried at 150 °C for one day. After drying process, a solid iron-promoted lignin mixture as a precursor was obtained. Then the iron-promoted lignin precursor was loaded in the middle of a 2-inch OD ceramic tubular reactor. The reactor was heated at a rate of 10°C /min to 1100 °C and kept at 1100 °C for 1 h with 50 mL/min argon and 50 mL/min methane. The furnace was naturally cooled down by 10 °C /min to room temperature. The few layers LG sample was collected from the 2-inch OD ceramic tubular reactor for further use.

For LGO and purification, a modified Hummers’ method was employed to oxidize LG and remove catalyst from LG to produce LGO [18]. Specifically, 200 mL 98% H_2_SO_4_, and 5.5 g NaNO_3_ were added into 2000 mL flask with 15 g LG, and the mixture was cooled down to 0 °C in an ice bath and stirred 10 min. Subsequently, 33 g KMnO_4_ was slowly added and continuously stirred for 30 min. Following this, 1000 mL DI water was added into the flask and the temperature was increased to 75 °C for 20 min. The mixture was then treated with 500 mL 10% H_2_O_2_ to terminate the chemical reaction. The total reaction time from LG to LGO was an hour which is noticeably less than that of graphene oxide made from natural graphite [19,20]. The LGO solution was washed by dialysis after centrifugation and sonication. The LGO powder can then be obtained from LGO solution after the freeze-drying process.

For the RLGO, a simple thermal annealing process was used to produce it from LGO powder. The RLGO powder was obtained by heating LGO powder in an oven at 200 °C in air for 2 h.

### 2.3. Scanning Electron Microscopy (SEM)

The morphology of the samples was investigated with a Scanning Electron Microscope (SEM). LG and LGO samples were mounted with conductive carbon tape, sputter coated with gold and imaged using field emission scanning electron microscope (LEO 1530 FESEM) at a 5 mm working distance and a 10 kV accelerating voltage.

### 2.4. Transmission Electron Microscopy (TEM)

The morphologies of LG and LGO samples were examined using a JEOL JEM-100CX II Transmission Electron Microscope (TEM, JEOL, Peabody, MA, USA) operated at an accelerating voltage of 200 kV. All TEM samples were sonicated in ethanol solution for 1 min before transfer to copper grids.

### 2.5. Atomic Force Microscopy (AFM)

The thickness of LGO samples were measured using atomic force microscopy (CS-3230, AFM workshop, Signal Hill, CA, USA). Samples were diluted to solids consistency of 0.05% and deposited onto clean mica substrates and air dried overnight at room temperature. The samples were imaged by a silicon cantilever in vibrating tapping mode at 160–225 kHz with a radium of the tip curvature less than 10 nm. The thickness and length of samples in the image were measured by Gwyddion software.

### 2.6. Thermogravimetric Analysis (TGA)

Thermal degradation and stability of Kraft lignin, LG, LGO, and RLGO was assessed by thermogravimetric analysis (TGA, PerkinElmer, Akron, OH, USA). Samples (1–3 mg) were heated from 50 °C to 800 °C with a ramp rate of 5 °C/min under a flowing nitrogen atmosphere (20 mL/min) by thermogravimetric analysis, respectively.

### 2.7. Wide Angle X-Ray Diffraction (XRD)

X-ray diffraction (XRD) patterns for LG, LGO and RLGO were obtained with a Bruker Discovery 8 diffractometer (Bruker, Madison, WI, USA) using a Cu Kα rotation tube at 50 kV and 1000 μA with scanning over the range of 2θ = 5–60°.

### 2.8. Raman Spectrum

Raman spectra of LG, LGO and RLGO were obtained by an Aramis Confocal Raman Microscope (HORIBA, Kyoto, Kyoto Prefecture, Japan) equipped with an excitation laser source emitting at 514 nm and having an incident power around 1 mW on a thin surface.

### 2.9. Fourier Transform Infrared Spectrum (FTIR)

The ATR-FTIR spectra of Kraft lignin, LG, LGO, and RLGO samples were recorded with the Thermo Scientific ATR-FTIR spectrometer (Thermo Scientific, Nicolet iZ10,Thermo Fisher, Waltham, MA, USA) at a resolution of 4 cm^−1^ for 64 scans in 500 to 4000 cm^−1^ range. All powdered samples were pressed against the diamond crystal of the ATR device. Background spectrum obtained by scanning the air was subtracted from the sample spectrum before being converted into transmittance units.

## 3. Results and Discussion

### 3.1. Morphologies

**Figure 1:**
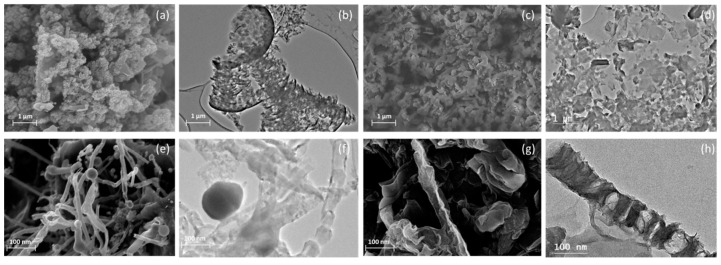
Morphologies of LG and LGO. (**a**,**b**) SEM and TEM images of flake-like LG; (**c**,**d**) SEM and TEM images of LGO nanoplatelet; (**e**,**f**) SEM and TEM images of tube-like LG, (**g**,**h**) SEM and TEM images of LGO nanotube.

Figure 1 shows microstructures and morphologies of SEM images and TEM images of LG after a catalytic graphitization process and LGO derived from LG by an oxidation reaction process, respectively. In Figure 1a,b, the images show the flake-like multi-layered LG. After oxidation reaction, the similar flake-like structure of LGO can be found in Figure 1c,d. For LG, another type of morphology, the tube-like structure is observed in SEM and TEM images of Figure 1e,f. Similarly, the same morphology of LGO nanotube structure is observed in Figure 1g,h which are derived from the tube-like LG, after oxidation reaction. It indicates that the oxidation reaction didn’t change the morphologies of LGO reacted from LG. Compared to graphene oxide derived from natural graphite, LGO has smaller nano scale lateral size (size less than 1 μm) than the micron scale graphene oxide commonly derived from natural graphite [21]. Moreover, the oxidation reaction time for LGO made from LG was in a hour, which is remarkably less than the oxidation reaction time of graphene oxide made from natural graphite, the oxidation reaction time of graphene oxide made from natural graphite generally takes a couple of hours [19,22,23]. The nano scale LGO might have unique properties to sufficiently fill some niche applications [24].

**Figure 2:**
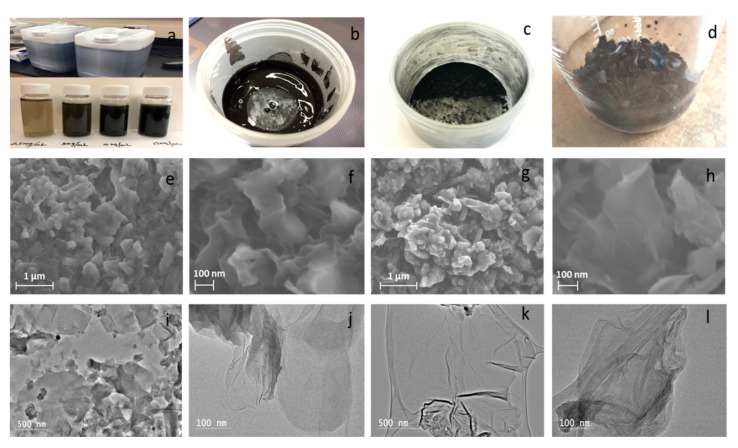
Synthesis of LGO aqueous solution, and solid nanoplatelets, and its morphology. (**a**) LGO aqueous solution. (**b**) LGO colloid. (**c**) LGO powders. (**d**) LGO slices. (**e**,**f**) SEM images of LGO aqueous solution. (**g**,**h**) SEM images of LGO nanoplatelets. (**i**,**j**) TEM images of LGO aqueous solution. (**k**,**l**) TEM images of LGO nanoplatelets.

Figure 2a–d shows LGO aqueous solution stored 1 year and LGO aqueous solutions with 0.5 mg/mL, 3 mg/mL, 10 mg/mL, and 15 mg/mL concentrations, LGO colloid, LGO powders and LGO slices, respectively. To observe the morphologies of LGO materials in different phases, SEM and TEM imaging was employed to observe the microstructures and morphologies of LGO in its aqueous and solid phase. Figure 2e,f shows SEM images targeting the morphology of LGO aqueous solution. Figure 2i,j shows the TEM images of LGO aqueous solution morphology. Figure 2g,h shows SEM images of LGO nanoplatelets and their morphology. Figure 2k,l shows the TEM morphology images of LGO nanoplatelets. These images (Figure 2g,h,k,l) show the LGO nanoplatelets in solid phase, stacked and wrinkled together, compared with the unfolded aqueous solution LGO in aqueous phase in (Figure 2e,f,l,j). The SEM images in Figure 2e–h show the nanoscaled flake-like LGO. This nanoscale lateral size LGO can also be distinctly observed in the TEM images of Figure 2i–l for both LGO aqueous solution and nanoplatelets.

### 3.2. FTIR Spectra

**Figure 3:**
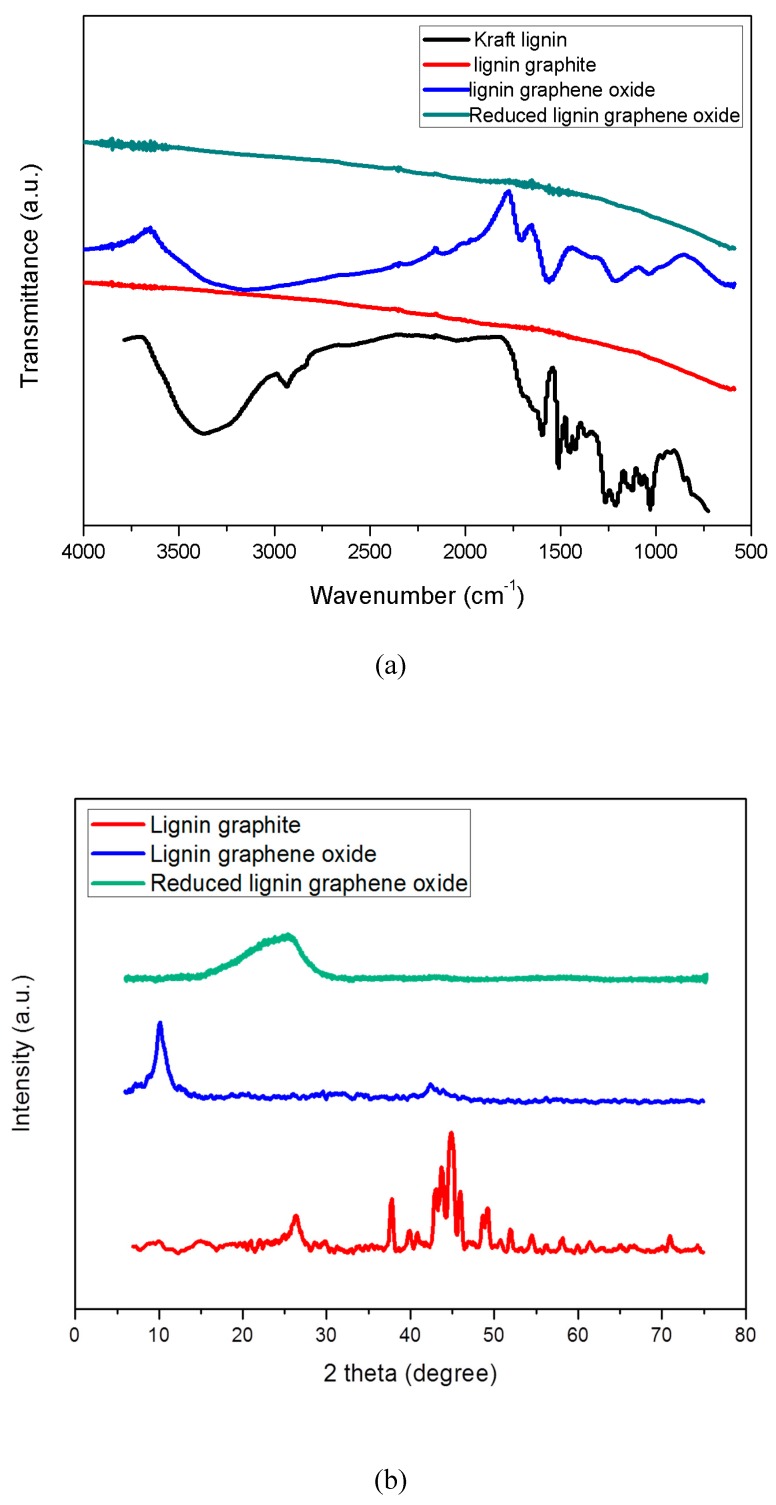

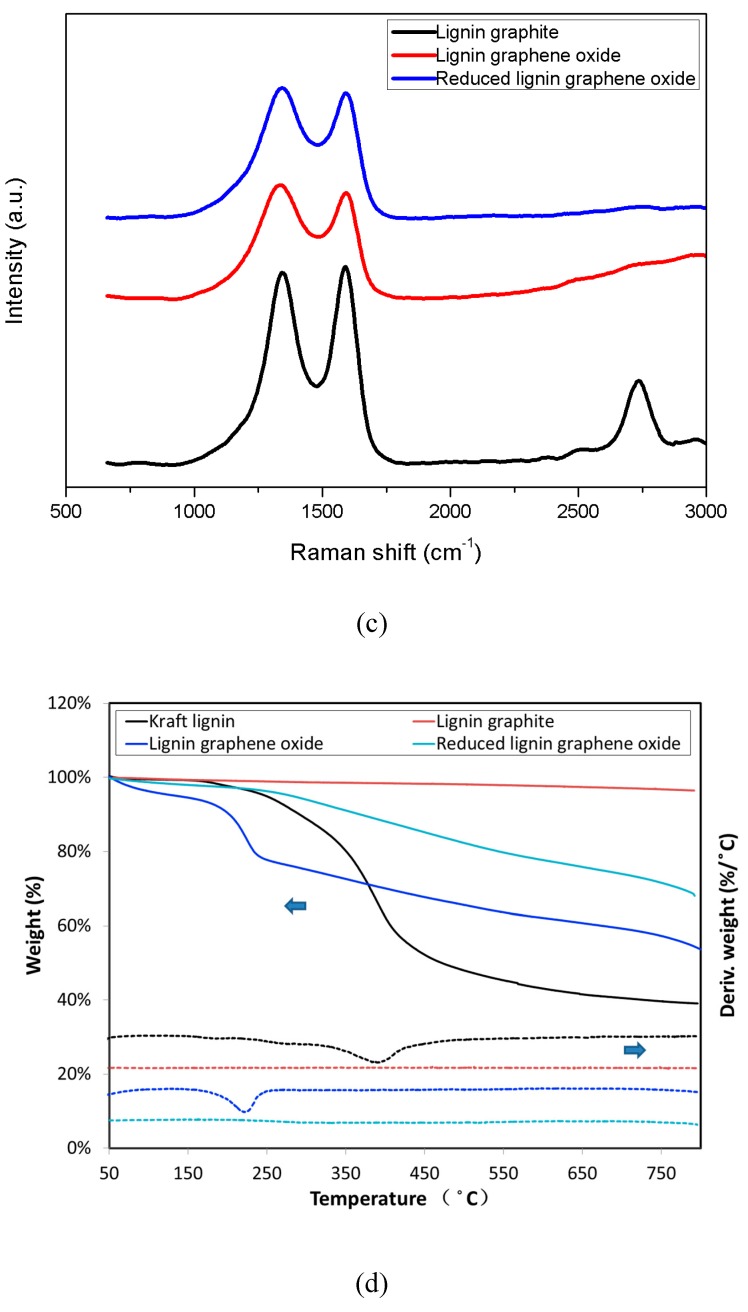

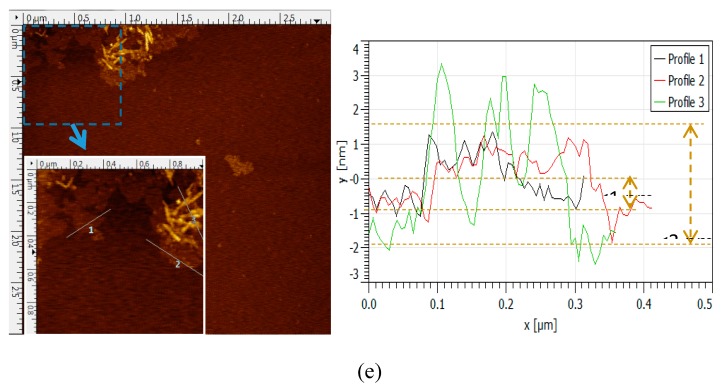
The properties and characteristics of LGO and its related materials. (**a**) FTIR spectra of Kraft lignin, LG, LGO, and RLGO; (**b**) XRD spectra of LG, LGO, and RLGO; (**c**) Raman spectra of LG, LGO, and RLGO. (**d**) TGA of Kraft lignin, LG, LGO, and RLGO. (**e**) Tapping mode AFM image of LGO and the thicknesses of LGO nanoplatelets measured from AFM image.

In Figure 3a, FTIR was used to measure the functional groups of Kraft lignin, LG, LGO and RLGO, respectively. The variation peaks and relative functional groups in the spectrum for Kraft lignin were discussed in our previous study [25]. After catalytic graphitization, the spectrum of LG shows no vibration peak, indicating all functional groups in this LG were completely removed by this thermal graphitization process due to the high temperature (Figure 3a). For LGO, the FTIR spectrum illustrates that oxygen groups were usefully added into the graphene structure, which identifies the same functional groups of graphene oxide made from natural graphite by oxidation reaction (Figure 3a): O-H stretching vibrations (3350 cm^−1^), C=O stretching vibrations (1734 cm^−1^), C=C from sp^2^ bonds (1615 cm^−1^), O–C–O vibrations (1238 cm^−1^) and C–O vibrations (1068 cm^−1^) [26]. However, the oxidation reaction time for LGO was remarkably reduced to one hour compared to the longer reaction time of graphene oxide made from natural graphite [27]. For RLGO, two weak vibration peaks around C=O stretching vibration (1734 cm^−1^) and C=C from sp^2^ bonds (1615 cm^−1^) were observed, and almost all functional groups had been removed through the thermal reduction process. It indicated that LGO can be successfully converted to RLGO through thermal reduction treatment.

### 3.3. XRD Patterns

In Figure 3b, X-ray diffraction (XRD) was performed on LG, LGO, and RLGO, respectively. The XRD pattern of Kraft lignin precursor with board diffraction peak at 21° was reported and discussed in our previous study [25]. The XRD pattern of the LG sample shows graphite peak at 26.55° corresponding to (002) plane, three γ-iron peaks at 43.5°, 50.6°, and 74.3° corresponding to (111), (200), and (220) planes, and the cementite peaks at 37.75°, 40.7°, 42.6°, 43.75°, 44.56°, 44.94°, 45.86°, 49.12°, and 57.8° corresponding to (121), (210), (201), (211), (102), (220), (031), (112), and (221) [14]. The results indicated that graphite structures were formed after thermal treatment at 1100 °C. The XRD pattern of the LGO sample shows a sharp peak around 11.2° corresponding to the (001) basal plane. This indicated that good oxidization and exfoliation of LG was achieved. Furthermore, no iron-based catalyst peaks can be observed in XRD pattern of LGO, it demonstrated that the catalyst has been removed in the reaction and the LGO sample has been purified without iron based catalyst after dialysis. The XRD pattern of RLGO exhibits a board peak at 25.6° corresponding to (002) plane, after thermal reduction of LGO, it was reduced to RLGO and the ordered crystal structure of RLGO was restored. In the experiments, dark brown suspension of LGO gradually transferred back into precipitate during thermal reduction, by observation.

### 3.4. Raman Spectra

Raman spectroscopy is a very important characterization tool to analyze carbon materials because Raman scattering has a close relationship with the electron structure of the substances. Figure 3d shows the Raman spectra of LG, LGO, and RLGO, respectively. It is well known that the G peak at 1580 cm^−1^ is the characteristic peak of sp^2^ hybrid structure, which represents the symmetry and crystallizability of graphene materials; and the D peak at 1350 cm^−1^ is the defect peak, which represents the surface defect and disorder of graphite layers [28,29]. In Figure 3d, the D peak of LGO and RLGO becomes stronger and broader due to a higher level of disorder among the graphene layers, and defects also seem to have increased during the oxidation process [30]. The 2D band at 2700 cm^−1^ of LG is a second order two phonon process [31], which means few-layer graphene. When graphene has less than 5 layers, a 2D band can be distinguished in Raman spectrum [32]. It indicated that the LG has few layers produced by catalytic graphitization process. The increase of I_D_/I_G_ from 1.0 of LG to 1.2 of LGO confirms the grafting of oxygen containing functional groups to the graphitic planes. After reduction treatment, I_D_/I_G_ of RLGO from 1.2 of LGO becomes 1.1, which indicates the removal of most of the oxygen containing functional group. The I_D_/I_G_ of RLGO is higher than that of LG due to the sp^2^ domains which are newly formed during reduction and smaller than those of LG [30].

### 3.5. TGA Measurement

In Figure 3d, thermogravimetric analysis (TGA) was performed on Kraft lignin, LG, LGO, and RLGO respectively. The result for Kraft lignin shows that the solids yield of Kraft lignin sample decreased from 98.5% to 51.2% as the temperature increased from 100 to 400 °C. The yield of Kraft lignin samples stayed around 40.1% as the temperature increased to 800 °C. The major Kraft lignin weight loss of 40.4% occurred as the temperature increased from 250 to 450 °C, and because of its thermal decomposition, the maximum weight loss is attained at 395 °C, shown in derivative thermogravimetric curve. The LG sample exhibits a high yield without noticeable degradation as the temperature increased to 800 °C, because the LG sample was well graphitized through the catalytic graphitization process, and only around 3.0% weight is lost from the LG sample as temperature increased to 800 °C in Figure 3d. The TGA curve of the LGO sample shows obvious mass loss below 200 °C, presumably attributed to the CO and CO_2_ streaming from the most labile functional group. The result of LGO exhibits another mass loss (28%) in the range of 220 to 800 °C, which has been attributed to the loss of oxygen functional groups. In contrast, the TGA result of the RLGO sample shows higher thermal stability than the LGO sample. The total loss is 27% at temperatures below 800 °C. This lower mass-loss can be attributed to the absence of most oxygen functional groups [33].

### 3.6. AFM Image and Thickness

Figure 3e shows the morphology and thickness of LGO samples by AFM. Figure 3e shows that LGO samples typically had flake-like and tube-like structures with less than 1 μm lateral size and the range of thickness of LGO samples was between 1 to 3 nm. The layer-to-layer distance (d-spacing) of graphene oxide is 0.850 nm [34], which indicated that this LGO sample typically has 1 to 3 layers. The lateral size of LGO was typically less than 1 μm, which was less than that of graphene oxide made from natural graphene. The sonication can exfoliate the LGO to fewer layers. The nanoscaled LGO may apply for conductive materials [24].

## 4. LGO Based Applications

**Figure 4:**
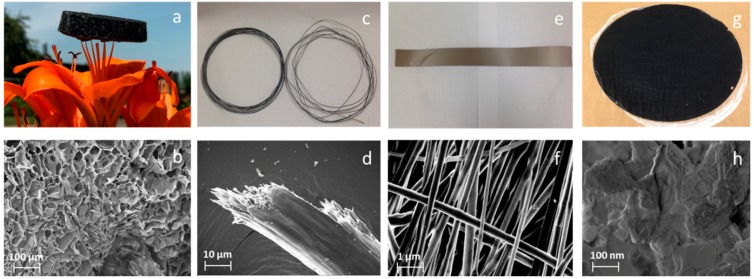
LGO based applications and their SEM images. (**a**,**b**) Ultra-light-weight LGO/CNF aerogel and its SEM image of microstructure; (**c**,**d**) electrical conductive LGO/(PAN) wire and its SEM image of microstructure; (**e**,**f**) electrospinning LGO/PAN fiber sheet and its SEM image of microstructure; (**g**,**h**) lignin graphene oxide/cellulose nanofibils film and its SEM image of microstructure.

The excellent dispersibility of LGO makes it a promising candidate to fabricate LGO based composite materials (For example, 1D fibers, 2D films, 3D framework structures). A 3D bio-aerogel prepared by a synergistic assembly of 50% LGO and 50% cellulose nanofibrils (CNF) through a freeze drying process shows the same appearance and internal structure as an assembly of prepared pure CNF aerogel reported by our previous research [35]. Results show the LGO/CNF aerogel had higher stiffness and strength than the pure CNF aerogel. In Figure 4c,d, LGO with polyacrylonitrile (PAN) with 1:1 by weight ratio was also used to fabricate continuous electrical wire by the wet-spinning method and thermal treatment. Dimethylformamide (DMF) solvent was used to dissolve the PAN and LGO mixture. It shows a uniform surface, similar to graphene oxide fibers made directly from undried graphene oxide suspensions. It might have better thermal conductivity due to the nano size nanoplatelets [24]. In Figure 4e,f, a LGO/PAN fiber sheet with 1:1 by weight ratio was fabricated by electrospinning process. Dimethylformamide (DMF) solvent was also used to dissolve the PAN and LGO mixture. The average fiber diameter was 200 nm, and the small size lignin graphene oxide nano platelets were very uniformly dispersed in the fiber sheet. In Figure 4g,h, a LGO/CNF film was made from the LGO and CNF solution with 1:1 weight ratio by the vacuum-assisted filtration method, which showed a well-aligned lamellar structure and comparable mechanical performance to pure CNF film.

## 5. Conclusions

The synthetic LGO and its relative materials produced from a new resource (Kraft lignin) have been established by catalytic graphitization, oxidation reaction and thermal reduction process. The experimental results obtained from XRD, FTIR, Raman, SEM, TEM and AFM showed this LGO has similar properties and characteristics to graphene oxide made from natural graphite, while the LGO has a one-hour oxidation reaction time that was remarkably less than that of graphene oxide made from natural graphite. Moreover, the nanoscaled lateral size of LGO that was observed by SEM, TEM and AFM was less than that of graphene oxide made from natural graphite, thus it could be used for some conductivity materials. In addition, the LGO has good dispersibility, and function groups of the LGO can be removed by thermal reduction processes to produce RLGO. Therefore, the LGO can be easily used to fabricate graphene based composites for various applications.
